# A debatable representation of all-cause mortality in Norway in 2024

**DOI:** 10.1177/14034948251393442

**Published:** 2026-01-07

**Authors:** Ann K. Skrindo Knudsen, Ingeborg Forthun, Christian Madsen, Marianne Strøm, Liv C. Vestrheim Thomsen, Jesper Dahl, Preben Aavitsland, Dagfinn Mørkrid Thøgersen, Hanne Løvdal Gulseth

**Affiliations:** 1 Department of Disease Burden, Norwegian Institute of Public Health, Bergen, Norway; 2 Department of Health Registry Research and Development, Norwegian Institute of Public Health, Bergen, Norway; 3 Centre for Cancer Biomarkers CCBIO, Department of Clinical Science, University of Bergen, Norway; 4 Department of Obstetrics and Gynaecology, Haukeland University Hospital, Bergen, Norway; 5 Department of Infection Control and Vaccines, Norwegian Institute of Public Health, Oslo, Norway; 6 Division for Infection Control, Norwegian Institute of Public Health, Oslo, Norway; 7 Pandemic Centre, University of Bergen, Norway; 8 Division of Public Health and Prevention, Norwegian Institute of Public Health, Oslo, Norway

**Keywords:** COVID-19, all-cause mortality, excess mortality

In their Short Communication, White et al. [1] make claims that, in our view, provide a debatable representation of mortality trends in Norway and the work done on mortality surveillance at the Norwegian Institute of Public Health (NIPH).

White et al. argue that NIPH’s approach for calculation of excess mortality in Norway for 2024 risks ‘incorrect conclusions if such a method is used to evaluate the current COVID-19 strategy’. This sentence contains an important ‘if’. The purpose of NIPH’s continuous surveillance of total mortality, including excess mortality, is to monitor and describe mortality trends in real time – not to evaluate health measures or pandemic strategies. NIPH has never claimed that the 2024 excess mortality estimates should be used to evaluate the Norwegian COVID-19 strategy.

Since SARS-CoV-2 is now among the viruses that affects mortality, it is necessary to use a model that accounts for this and other recent general mortality trends in routine surveillance. In line with European monitoring of excess mortality (EuroMOMO) [2], we therefore include 2023 in the baseline for weekly expected mortality in 2024, and 2023 and 2024 in the baseline for 2025. We have previously clearly described the change in baseline and model, and the rationale behind this change [3,4]. However, weekly spikes might not necessarily be of a size or a duration that triggers alerts of excess mortality. Further, cumulative annual totals can reveal longer-term trends in mortality. For complementary perspectives, we have therefore also published estimates on excess deaths for the full year of 2024, based on a summary of the estimated weekly excess deaths throughout 2024 [3]. By using the same model for the annual report as in routine surveillance, we ensure consistency in the estimates of weekly and annual excess mortality.

Using the baseline that included weekly observed age-standardized mortality rates in 2011–2019 and 2023, we estimated 1415 extra deaths in 2024. However, as this number was within the prediction interval of expected deaths, we concluded that there was no statistically significant excess mortality in 2024 [3]. White et al. estimated 2898 excess deaths using what they call their conservative approach. Why the discrepancy between these numbers? In the conservative approach of White et al., the observed mortality rate in 2023 was not used to inform expected mortality in 2024. Instead, the predicted value for 2023 was carried forward. This implies that their 2024 estimate, indirectly, is also based on a baseline extending only up to 2019. This model, and the interpretation of the results from it, rests on a strong assumption that mortality during 2020–2024 can be fully predicted from 2010–2019 annual data, and that only the pandemic influenced mortality in this period.

In 2023 and 2024, the age-standardized mortality rate (shown not in White and colleagues’ paper, but in Figure 1 in Forthun et al. [3]) returned to pre-pandemic levels. Further, from 2023 to 2024, there was a substantial decline in the mortality rates in younger age groups (0–19 and 20–39 years). Although changes in mortality rates cannot be used directly to measure change in excess mortality, the observed trends in the rates might indicate a temporary peak in pandemic-related mortality in Norway consistent with patterns seen in other countries [2]. A longer period of observation is necessary to determine whether this decrease in mortality rates is a persistent trend.

We question the claim of White et al. that their findings align with broader Nordic patterns of ‘disrupted mortality decline across Denmark, Finland, and Sweden since the post-acute pandemic phase, with all countries showing downward shifts in life expectancy’. The study they cite [5] includes only people aged 70 and older and ends in 2022. It therefore cannot substantiate their broader claim of a disrupted mortality decline across the Nordic populations in more recent years. Excluding younger age groups and omitting post-2022 data limits the reference’s relevance, particularly as more recent reports suggest a return toward pre-pandemic life expectancy levels [6,7].

Despite lacking data on causes of death, White et al. further suggest that the ‘unmitigated spread of SARS-CoV-2 in Norway since 2022 can be associated with increased mortality, particularly for those under 65’. The important word in this sentence is ‘can’, making it clear that this is a hypothesis, not a finding. When brought into journal publication and potentially public discourse, such nuances are sadly often missed. For younger age groups, where deaths are very few [8], annual fluctuations are expected. Small absolute changes in all-cause or cause-specific deaths can produce large relative differences, making consistent trends difficult to detect. Caution is therefore warranted when interpreting annual changes in mortality for younger populations and hypothesizing about their cause.

In conclusion, there is no universally accepted method for calculating excess mortality, and differences in estimates from alternative methods need to be interpreted alongside their underlying assumptions. White et al. make claims that we consider debatable and, in our view, misrepresent the post-pandemic mortality trends in Norway. Importantly, attributing excess mortality directly to the unmitigated spread of SARS-CoV-2 virus lacks support in available data and overlooks alternative explanations.
